# Revolutionizing Maxillary Rehabilitation: Zygomatic Implants Addressing Severe Alveolar Atrophy

**DOI:** 10.7759/cureus.61430

**Published:** 2024-05-31

**Authors:** Arushi Beri, Sweta G Pisulkar, Bhushan Mundada, Akansha Bansod, Shruti Deshmukh, Pooja Bhardwaj

**Affiliations:** 1 Prosthodontics, Sharad Pawar Dental College and Hospital, Wardha, IND; 2 Prosthodontics, Acharya Vinoba Bhave Rural Hospital, Wardha, IND; 3 Prosthodontics and Crown & Bridge, Sharad Pawar Dental College and Hospital, Wardha, IND; 4 Oral and Maxillofacial Surgery, Sharad Pawar Dental College and Hospital, Wardha, IND; 5 Periodontics, Rishiraj College of Dental Science, Bhopal, IND

**Keywords:** endosteal implants, maxillary atrophy, fp 1 prosthesis, immediate temporisation, zygomatic implants

## Abstract

This case report describes the care given to a 58-year-old male patient with severe upper jaw atrophy. The treatment strategy involved utilizing zygomatic implants in conjunction with endosteal implants to rehabilitate both the maxilla and mandible. Temporary prostheses were used during the healing phase, followed by the fabrication and placement of final prostheses. The utilization of zygomatic implants offers advantages such as immediate stabilization and function without the need for extensive bone grafting. This approach not only reduces treatment time and costs but also enhances patient outcomes. Furthermore, guided surgical techniques are increasingly employed to ensure precise implant placement, optimizing prosthetic support.

## Introduction

In typical dental implant treatments, managing severely resorbed partially or fully edentulous maxillae that are worsened by osseous resorption and maxillary sinus expansion poses significant complications. These challenges often result in prolonged treatment duration, increased morbidity, and higher costs for patients. However, the zygomatic process can now be used for quick implant stabilization and fixation with immediate function since other therapeutic options have emerged. As a result, longer-term grafting is not necessary, and the implant can be positioned in denser, more stable bone [1-4].

Zygomatic implants have emerged as a valuable solution over the past two decades for addressing severely resorbed maxillae. Initially introduced by Brånemark in 1988 and subsequently made available for clinical use, early protocols encountered complications primarily due to implant positioning. However, advancements in implant design and surgical techniques have effectively mitigated these issues [5].

In situations where numerous grafting surgeries are not practical or feasible and maxillary bone volume is insufficient, particularly in certain areas like the paranasal or lateral pyriform border, zygomatic implants offer a promising solution for immediate stabilization and functional restoration. These implants, varying in length from 30 mm to 60 mm and diameter from 3.5 mm to 4.5 mm, are designed to engage the zygoma at an angle, requiring angled corrections at their platforms to accommodate the trajectory through the maxillary sinus [6].

Classification schemes such as the Aparicio classification are necessary due to anatomical differences and help classify zygomatic implants according to where they are in relation to the sinus's lateral wall. A portion of the implants pass through the sinus, especially along the outside of the lateral wall, without being covered by bone. Their length guarantees stability in the zygoma's dense bone [7-11].

Incorporating zygomatic implants into severely resorbed maxillae can be integrated into conventional "All-on-4" procedures or expanded to quad zygoma procedures in cases where the paranasal region cannot accommodate anterior implant fixtures. Furthermore, zygomatic implants serve as a viable alternative for partially edentulous maxillae with insufficient bone volume, eliminating the need for osseous grafting and thereby reducing treatment time and complexity [12-16].

## Case presentation

**Figure 1:**
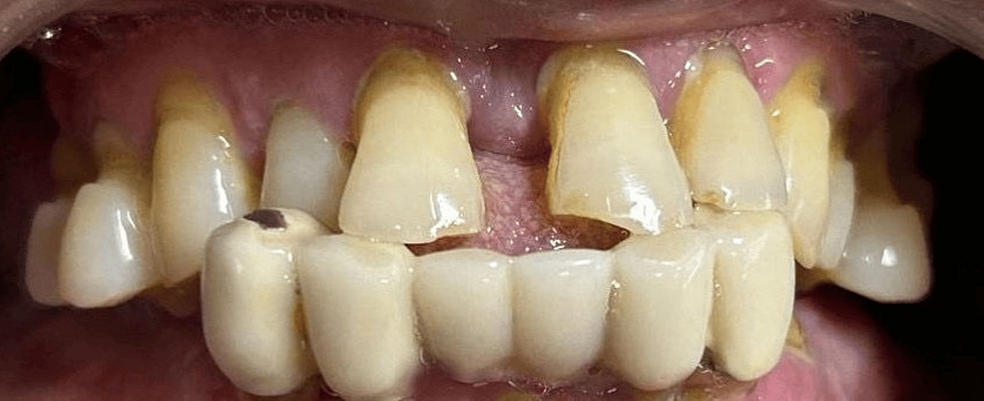
Pre-operative intraoral examination

**Figure 2:**
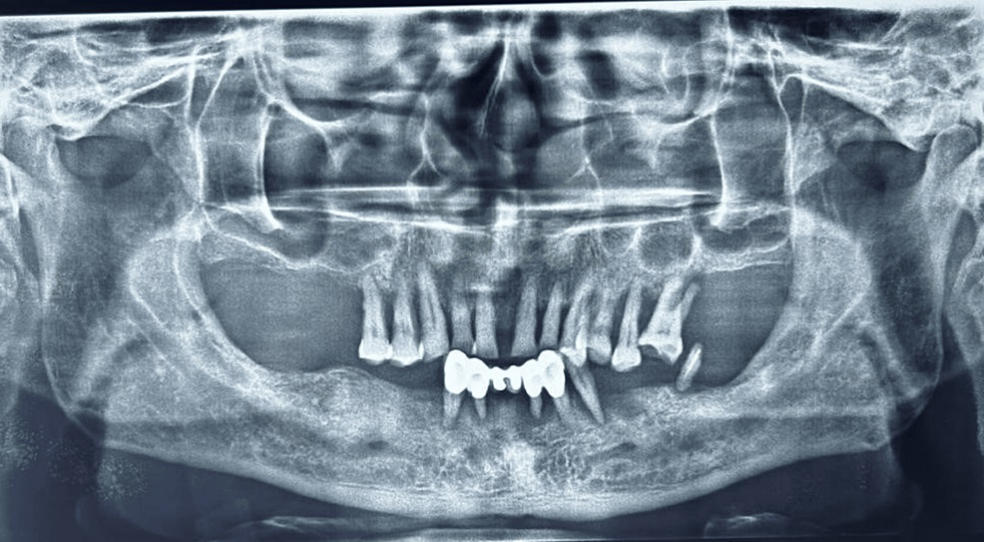
Pre-operative radiographic evaluation

The article describes a case that records the recovery process for a 58-year-old male patient suffering from significant maxillary atrophy. The patient presented to the department with the complaint of having trouble speaking and mastication for eight years due to numerous missing, carious, and movable teeth in his mandibular and maxillary arches. The aesthetic appearance of a dental restoration was significantly compromised, resulting in a reduction in the patient's confidence level. Upon intraoral examination, it was noted that teeth 15 through 26 in the maxillary arch exhibited grade 2 mobility, while teeth 33 through 43 in the mandibular arch displayed a fixed prosthesis with grade 3 mobility, with teeth 34 and 37 root pieces also showing mobility (Figure 1). Radiographic evaluation revealed crown root ratio throughout the dentition and severe atrophy in the maxillary posterior region (Figure 2).

**Figure 3:**
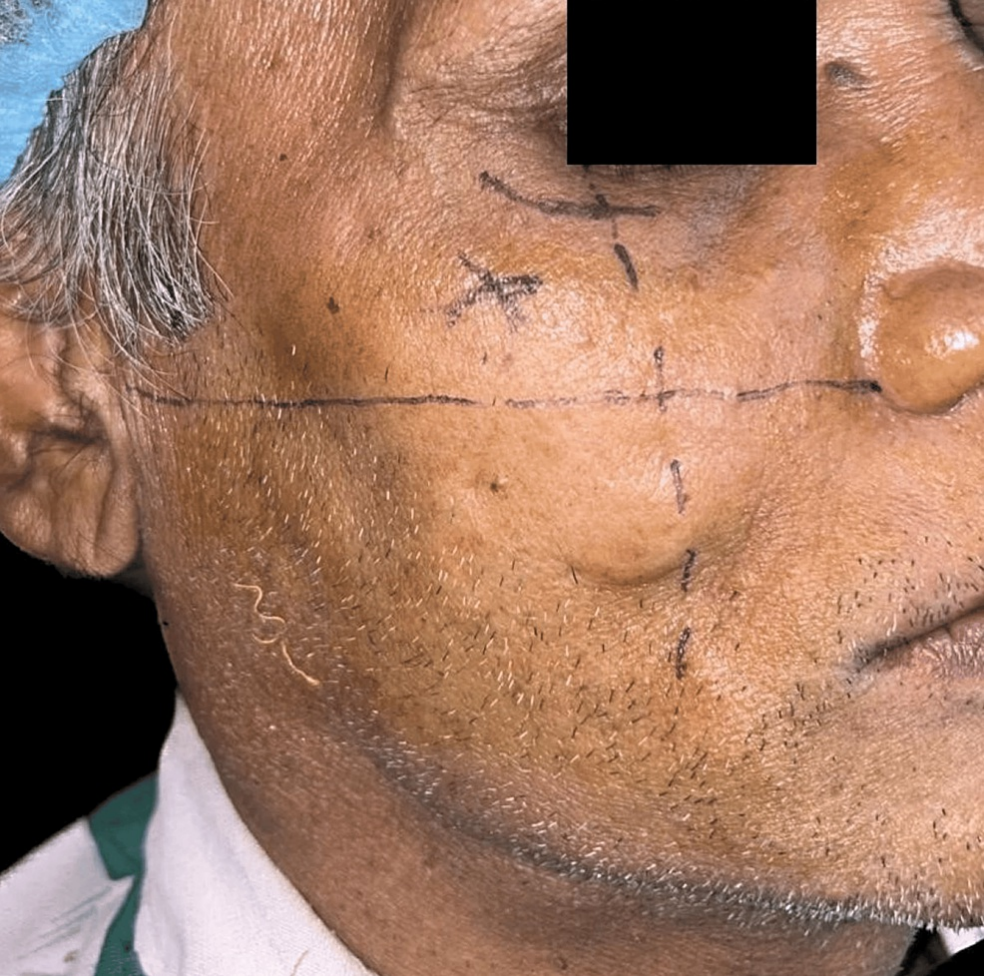
Extraoral markings for placement of zygomatic implants

**Figure 4:**
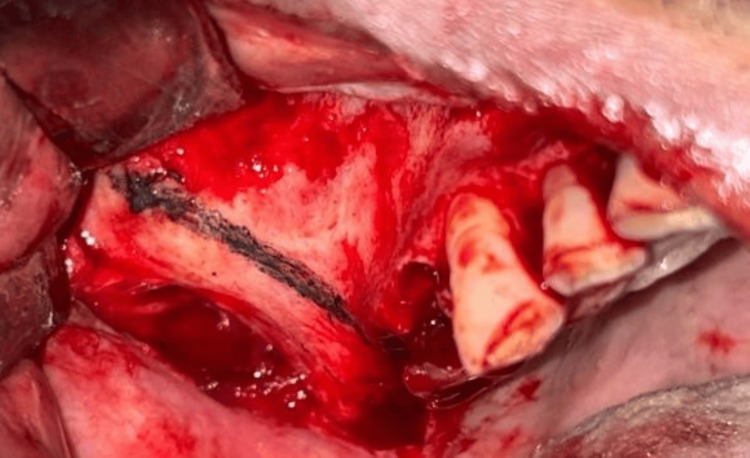
Intraoral marking for osteotomy preparation

**Figure 5:**
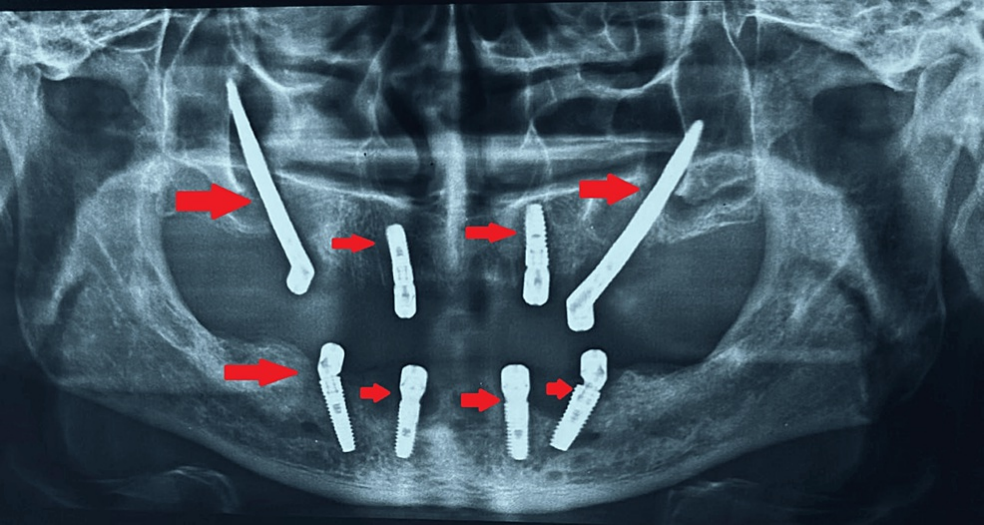
Post-implant placement radiograph Arrows are indicative of implant position.

**Figure 6:**
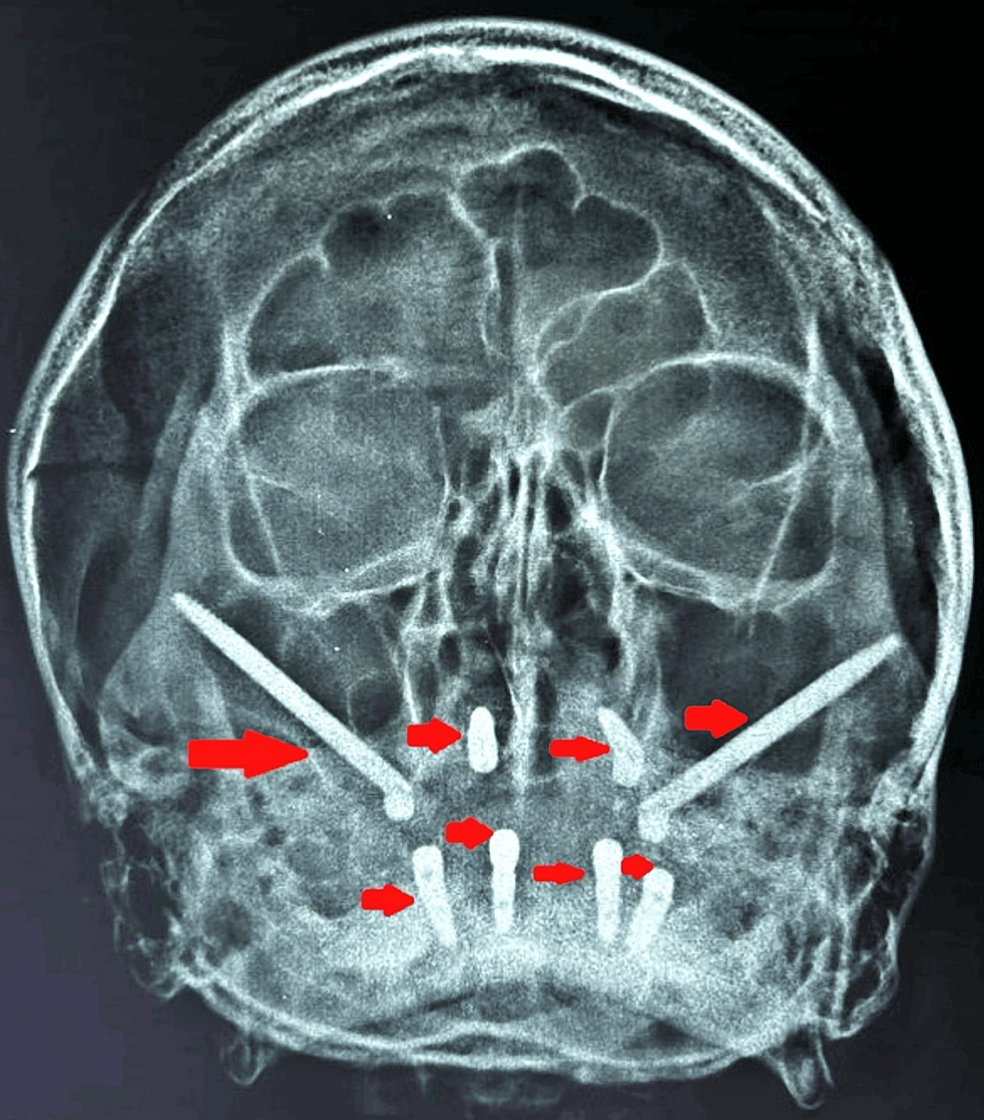
Waters view projection showing implant position Arrows are indicative of implant position

Various diagnostic procedures, including blood tests (BT, CT RBS, HbA1C, hb%), and blood pressure evaluation, were conducted during the pre-surgical phase. Following consultation with a physician, the patient was scheduled for implant placement under local anesthesia. During the surgical phase, meticulous aseptic precautions were observed. Extraoral markings were made to guide the zygomatic bone approach (Figure 3), while intraoral markings were used for flap reflection, allowing for a direct approach to the zygomatic bone and for osteotomy preparation (Figure 4). Sequential drilling was performed to create osteotomy sites for implant placement. This included the placement of zygomatic implants sized 4.2 x 60 mm in the 15 regions and 4.2 x 62 mm in the 25 regions, as well as two endosteal implants sized 4.5 x 13 mm in the maxillary anterior region and similarly sized endosteal implants in the mandibular anterior and posterior regions. Hemostasis was achieved, sutures were applied, and post-operative medications were administered. The patient was monitored at intervals of 3, 6, 8, and 12 hours post-surgery, and radiographs were taken in order to ensure the proper position of implants as shown in Figures 5-6. Delayed loading was done.

**Figure 7:**
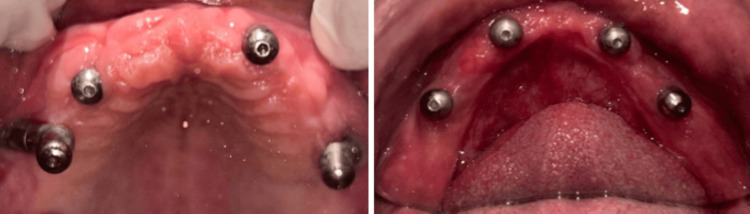
Completely healed mucosa with healing caps seen in the maxillary and mandibular arches respectively

**Figure 8:**
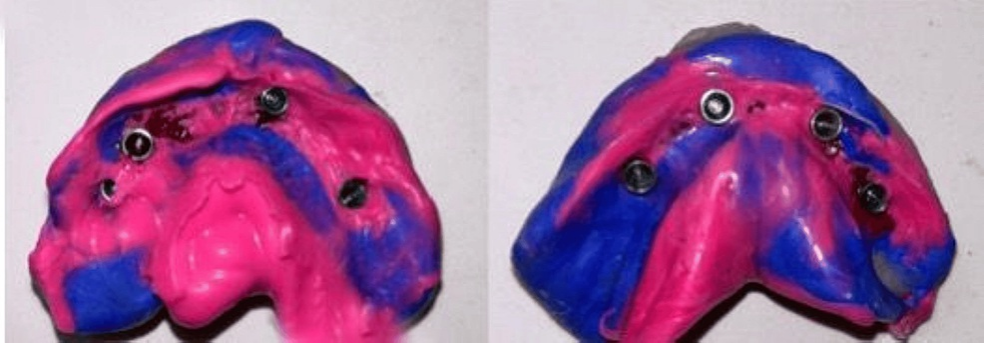
Open-tray impressions of the maxillary and mandibular arches made with putty and light body

**Figure 9:**
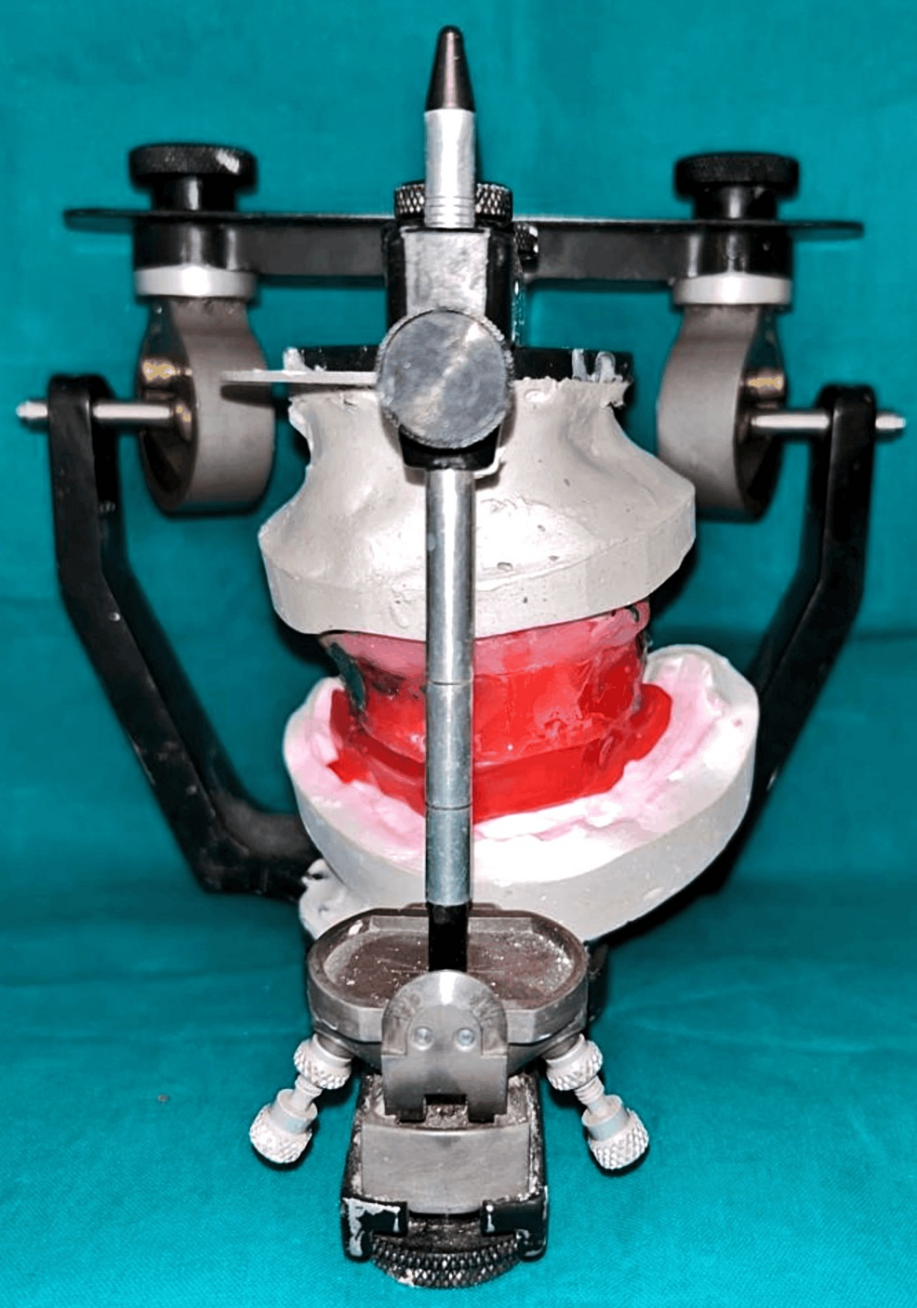
Facebow transfer on Hanau wide-vue articulator

**Figure 10:**
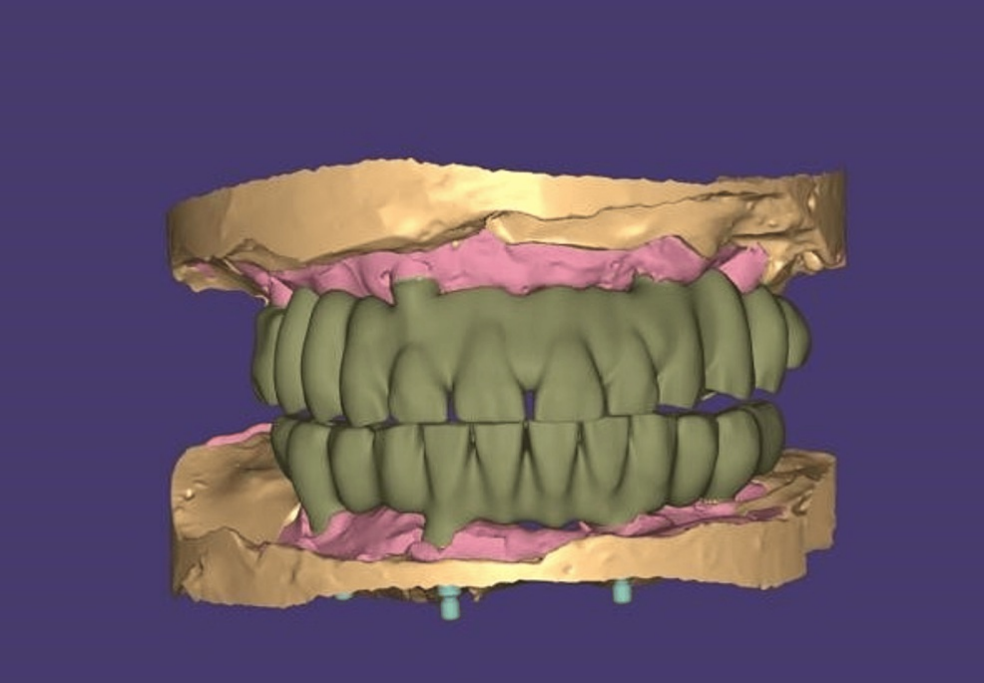
Designing of metal framework digitally using exocad software

**Figure 11:**
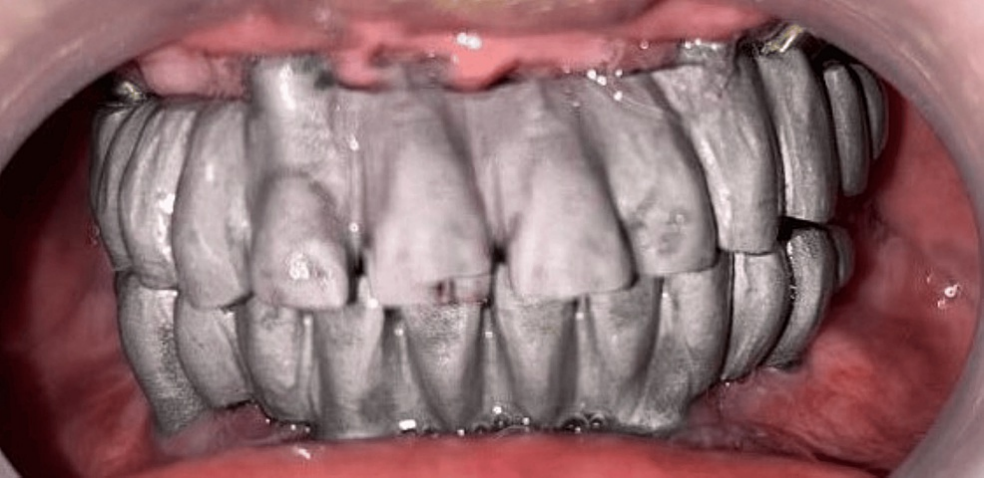
Metal framework try-in

**Figure 12:**
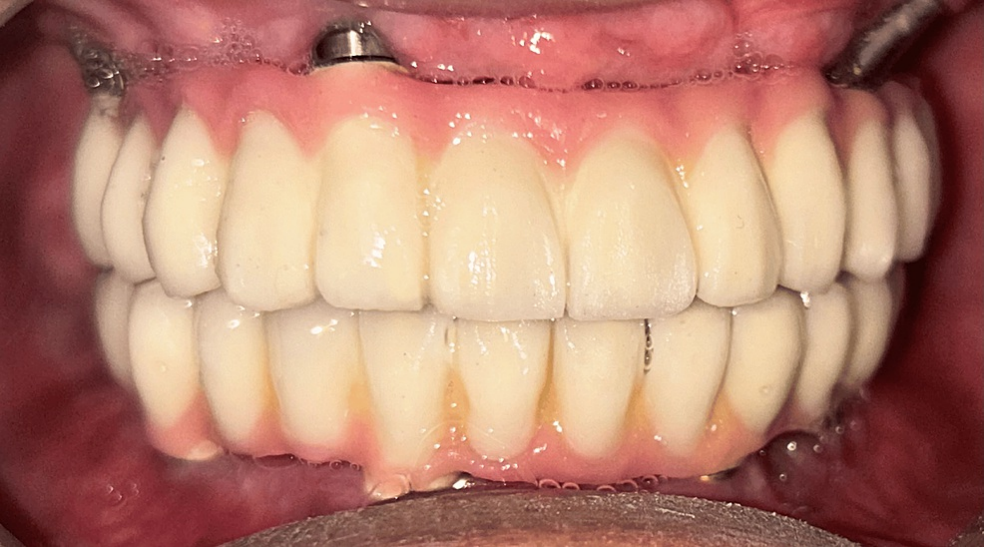
Final prosthesis post cementation

**Figure 13:**
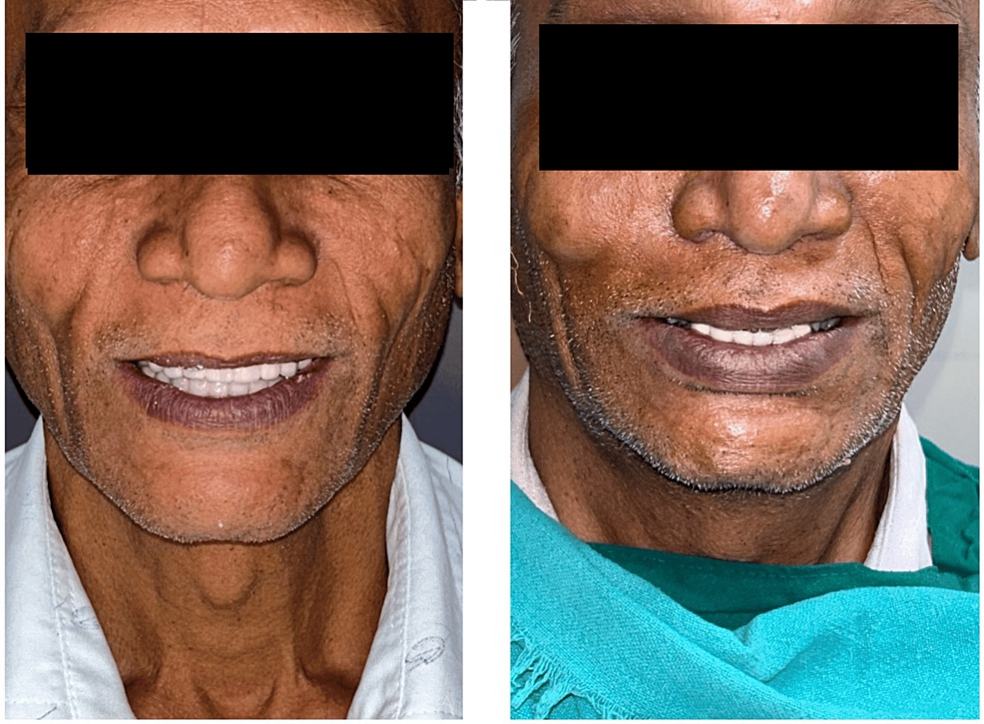
Comparison of pre- (L) and post-operative (R) photographs

The completely healed mucosa is shown in Figure 7. Three months after implant placement, the patient underwent a recall visit for open tray impression making. This involved joining impression copings with pattern resin and utilizing light body putty impression material as shown in Figure 8. A fabrication jig was then created, followed by a jig trial and jaw relation assessment, and then a facebow record was made and transferred to a Hanau wide-vue articulator (Whip Mix Corp., Louisville, KY) again to accommodate post-surgical changes in the vertical dimension as shown in Figure 9. Wax try-in was performed to assess occlusal interferences, followed by the designing of a metal framework digitally using exocad (exocad GmbH, Darmstadt, Germany) as shown in Figure 10, and fabrication of a metal framework using direct metal laser sintering (DMLS) technology (Figure 11). The metal framework was layered with porcelain of appropriate shade, and a bisque trial was conducted before the final finishing and polishing of the prosthesis. Final prosthesis insertion was performed (Figure 12), with subsequent follow-up visits scheduled after one week, three months, and six months. A comparison of the pre- and post-operative images is shown in Figure 13.

## Discussion

In the posterior maxilla, bone loss, increased expansion of the maxillary sinus, and decreased bone quality frequently make conventional implant placement difficult; it also needs significant osseous grafting. To increase bone volume for supporting conventional implants, a number of bone augmentation procedures have been described, including onlay grafting and sinus augmentation using lateral and crestal methods. However, these approaches may extend treatment duration and escalate associated costs. To address these challenges, alternative strategies to grafting procedures in the atrophic maxilla have been explored [17,18].

Zygomatic implants offer a potential treatment for individuals with severely resorbed totally or partially edentulous maxillary arches. They have been used in clinical therapy for the past 20 years. However, there are hazards associated with using zygomatic implants, and their insertion requires surgical competence. In the literature, sinusitis is the most often documented complication, with an incidence as high as 26.6%. Severe sinusitis difficulties are not usually linked to zygomatic implant placement, yet sinus issues may be predicted given that part of the implant passes into the maxillary sinus. Postoperative hemorrhage, vestibular cortical fenestration, oroantral fistula formation, and transient sensory nerve impairment are other possible problems [19-22].

Individuals who have prominent buccal concavities on the lateral aspect of the maxillary sinus may be more susceptible to problems, particularly if they undergo non-guided surgery. This technique is clinically challenging due to the anatomical complexity of the structures and the complexities of the zygoma [23-25]. On the other hand, new developments in technology, like virtual implant planning, cone beam computed tomography (CBCT), and computer-aided design/computer-aided manufacture (CAD/CAM) surgical guide fabrication, have the potential to lower problems and increase placement accuracy.

Numerous studies have indicated good success rates with zygomatic implants, despite some problems. Positive long-term results have been obtained, with cumulative success rates between 97% and 100%. Following a 36-month follow-up, a systematic evaluation of research published between 2000 and 2012 found a 97.86% survival rate. The American College of Prosthodontists recognizes the adaptability of zygomatic implants in a range of therapeutic contexts, emphasizing their contribution to improving patients' quality of life and offering effective and dependable treatment alternatives [25-31].

## Conclusions

In conclusion, zygomatic implants emerge as a promising alternative in the management of severely atrophic fully or partially edentulous maxillae, offering a valuable option alongside traditional osseous grafting for implant placement. Their comparable success rates and survival outcomes to conventional implants hint at the potential to streamline treatment protocols, thereby reducing both the time and financial burdens associated with complex grafting procedures. With advancing technologies like cone beam computed tomography (CBCT), virtual planning, and surgical guides, the utilization of zygomatic implants is poised to grow, promising a future marked by fewer complications and enhanced patient care. This research underscores the importance of integrating innovative solutions in dental practice to improve patient outcomes and optimize clinical workflows.

## References

[1] Beri A, Pisulkar SG, Mundada BP, Borle A, Dahihandekar C, Bansod A (2023). Quad zygoma: A graftless solution in post-mucormycosis maxillectomy. Cureus.

[2] Wessberg GA, Jacobs MK, Wolford LM, Walker RV (1982). Preprosthetic management of severe alveolar ridge atrophy. J Am Dent Assoc.

[3] Misch CE (1987). Maxillary sinus augmentation for endosteal implants: organized alternative treatment plans. Int J Oral Implantol.

[4] Lamberti VS (1994). Subantral graft: clinical application of the biological principles osseoinduction in the treatment of posterior maxillary atrophy [article in Spanish]. Int J Dent Symp.

[5] Chanavaz M, Donazzan M, Ferri J, Tatum H, Francke JP, Fenart R (1995). Sinus augmentation. Statistical evaluation of 15 years of surgical experience (Manuel Chanavaz, 1979-1994) [article in Spanish]. Rev Stomatol Chir Maxillofac.

[6] Urban IA, Monje A, Lozada JL, Wang HL (2017). Long-term evaluation of peri-implant bone level after reconstruction of severely atrophic edentulous maxilla via vertical and horizontal guided bone regeneration in combination with sinus augmentation: a case series with 1 to 15 years of loading. Clin Implant Dent Relat Res.

[7] Moreno Vazquez JC, Gonzalez de Rivera AS, Gil HS, Mifsut RS (2014). Complication rate in 200 consecutive sinus lift procedures: guidelines for prevention and treatment. J Oral Maxillofac Surg.

[8] Petrungaro PS (2000). Reconstruction of severely resorbed atrophic maxillae and management with transitional implants. Implant Dent.

[9] Maló P, Rangert B, Nobre M (2005). All-on-4 immediate-function concept with Brånemark System implants for completely edentulous maxillae: a 1-year retrospective clinical study. Clin Implant Dent Relat Res.

[10] Wallace SS, Froum SJ (2003). Effect of maxillary sinus augmentation on the survival of endosseous dental implants. A systematic review. Ann Periodontol.

[11] Jensen OT, Cottam JR, Ringeman JL, Graves S, Beatty L, Adams MW (2014). Angled dental implant placement into the vomer/nasal crest of atrophic maxillae for All-on-Four immediate function: a 2-year clinical study of 100 consecutive patients. Int J Oral Maxillofac Implants.

[12] Maló P, Nobre Mde A, Lopes I (2008). A new approach to rehabilitate the severely atrophic maxilla using extramaxillary anchored implants in immediate function: a pilot study. J Prosthet Dent.

[13] Bedrossian E, Stumpel LJ 3rd (2001). Immediate stabilization at stage II of zygomatic implants: rationale and technique. J Prosthet Dent.

[14] Aparicio C, Ouazzani W, Hatano N (2008). The use of zygomatic implants for prosthetic rehabilitation of the severely resorbed maxilla. Periodontol 2000.

[15] Aparicio C (2011). A proposed classification for zygomatic implant patient based on the zygoma anatomy guided approach (ZAGA): a cross-sectional survey. Eur J Oral Implantol.

[16] Aboul-Hosn Centenero S, Lázaro A, Giralt-Hernando M, Hernández-Alfaro F (2018). Zygoma quad compared with 2 zygomatic implants: A systematic review and meta-analysis. Implant Dent.

[17] Malevez C (2012). Zygomatic anchorage concept in full edentulism [article in French]. Rev Stomatol Chir Maxillofac.

[18] Chrcanovic BR, Abreu MH (2013). Survival and complications of zygomatic implants: a systematic review. Oral Maxillofac Surg.

[19] Beri A, Pisulkar SK, Bansod AV, Godbole S, Shrivastava A (2023). Rehabilitation of edentulous patient with customized functional palatal contours. J Datta Meghe Inst Med Sci Univ.

[20] Esposito M, Worthington HV, Thomsen P, Coulthard P (2003). Interventions for replacing missing teeth: dental implants in zygomatic bone for the rehabilitation of the severely deficient edentulous maxilla. Cochrane Database Syst Rev.

[21] Molinero-Mourelle P, Baca-Gonzalez L, Gao B, Saez-Alcaide LM, Helm A, Lopez-Quiles J (2016). Surgical complications in zygomatic implants: A systematic review. Med Oral Patol Oral Cir Bucal.

[22] Araújo RT, Sverzut AT, Trivellato AE, Sverzut CE (2017). Retrospective analysis of 129 consecutive zygomatic implants used to rehabilitate severely resorbed maxillae in a two-stage protocol. Int J Oral Maxillofac Implants.

[23] Fernández H, Gómez-Delgado A, Trujillo-Saldarriaga S, Varón-Cardona D, Castro-Núñez J (2014). Zygomatic implants for the management of the severely atrophied maxilla: a retrospective analysis of 244 implants. J Oral Maxillofac Surg.

[24] Tzerbos F, Bountaniotis F, Theologie-Lygidakis N, Fakitsas D, Fakitsas I (2016). Complications of zygomatic implants: our clinical experience with 4 cases. Acta Stomatol Croat.

[25] Pathak A, Dhamande MM, Sathe S, Gujjelwar S, Khubchandani SR, Minase DA (2023). Unveiling the realm of denture fabrication: revitalizing aesthetics and optimizing efficiency for geriatric patients. Cureus.

[26] D'Agostino A, Trevisiol L, Favero V, Pessina M, Procacci P, Nocini PF (2016). Are zygomatic implants associated with maxillary sinusitis?. J Oral Maxillofac Surg.

[27] Beri A, Pisulkar SK, Paikrao B, Bagde A, Bansod A, Shrivastava A, Jain R (2024). Quantitate evaluation of photogrammetry with CT scanning for orbital defect. Sci Rep.

[28] Bothur S, Kullendorff B, Olsson-Sandin G (2015). Asymptomatic chronic rhinosinusitis and osteitis in patients treated with multiple zygomatic implants: a long-term radiographic follow-up. Int J Oral Maxillofac Implants.

[29] Beri A, Pisulkar SK, Bansod AV, Shrivastava A, Jain R (2023). Tissue engineering in maxillofacial region from past to present. J Datta Meghe Inst Med Sci Univ.

[30] Pathak A, Dhamande MM, Sathe S, Gujjelwar S (2023). Effectiveness, esthetics, and success rate of dental implants in bone-grafted regions of cleft lip and palate patients: a systematic review and meta-analysis. Cureus.

[31] Polido WD, Machado-Fernandez A, Lin WS, Aghaloo T (2023). Indications for zygomatic implants: a systematic review. Int J Implant Dent.

